# Ki67 is a biological marker of malignant risk of gastrointestinal stromal tumors: A systematic review and meta-analysis

**DOI:** 10.1097/MD.0000000000007911

**Published:** 2017-08-25

**Authors:** Yu Zhou, Wenqing Hu, Ping Chen, Masanobu Abe, Lei Shi, Si-yuan Tan, Yong Li, Liang Zong

**Affiliations:** a Department of General Surgery, Suzhou Municipal Hospital (North Campus), Suzhou, Jiangsu Province, China; b Department of Gastrointestinal Surgery, Clinical Medical College of Yangzhou University (the Northern Jiangsu People's Hospital), Yangzhou, Jiangsu Province, China; c Department of Surgery, Heji Hospital Affiliated to Changzhi Medical College, Changzhi, China; d Division for Health Service Promotion, University of Tokyo, Tokyo, Japan; e Department of Gastrointestinal Surgery, Graduate School of Medicine, University of Tokyo, Tokyo, Japan.

**Keywords:** gastrointestinal stromal tumors, Ki-67, malignant risk, meta-analysis

## Abstract

**Background:**

Ki67 is a good marker of cell proliferation in a variety of tumors. High ki67 levels are usually associated with poor prognosis. However, the relationship between Ki67 expression and the risk of malignancy of gastrointestinal stromal tumors (GISTs) is still poorly defined. The current meta-analysis was initiated to address this issue.

**Methods:**

Studies reporting Ki67 expression and the risk of malignancy in GIST were found by searching Cochrane Library, PubMed, Medline, and Embase until October 31, 2016. A total of 9 studies involving 982 patients were included. Pooled odds ratio (OR) estimates and 95% confidence intervals (CIs) were calculated using a fixed-effect model.

**Results:**

Meta-analysis showed no significant difference in the incidence of Ki67 overexpression between the very low NIH group and the low NIH group (OR: 0.66, 95% CI: 0.25–1.76; *P* = .41, *P*_heterogeneity_ = .25). However, the incidence of Ki67 overexpression gradually increased from the low NIH group to the high NIH group (OR: 0.46, 95% CI: 0.27–0.80; *P* = .005, *P*_heterogeneity_ = .13) and (OR: 0.22, 95% CI: 0.15–0.34; *P* < .00001, *P*_heterogeneity_ = .33).

**Conclusions:**

There were more GIST patients with Ki67 overexpression in the intermediate and high NIH groups than in the low NIH group. Ki67 overexpression may be a useful marker of the risk of malignant GIST transformation.

## 1 Introduction

Gastrointestinal stromal tumor (GIST) is the most common mesenchymal tumor of the gastrointestinal (GI) system. Its annual incidence may range from 6.8 to 19.7 per million worldwide.^[1–3]^ GIST is known for its wide variety of biological behaviors and its difficult-to-predict potential malignancy.^[4,5]^ There has been a great deal of research into many aspects of GIST's biological behavior during the past decade.

Numerous studies have used correlations among biological behaviors to predict the prognosis of GIST, including mitotic count,^[6]^ tumor size,^[7]^ KIT mutations,^[8]^ predominant cell type,^[9]^ cellular density,^[10]^ p53,^[11]^ and other factors. In 2001, GIST was categorized by National Institutes of Health (NIH) into 4 groups on the basis of the 2 parameters, tumor size and mitotic count: very low risk, low risk, intermediate risk, and high risk (Table 1).^[12]^ Three years later, tumor site was added to the NIH system, which was then called the National Comprehensive Cancer Network (NCCN) criteria, based on Miettinen and Lasota's^[13]^ Armed Forces Institute of Pathology (AFIP) system.^[14]^ Till now, National Institutes of Health (NIH) and National Comprehensive Cancer Network (NCCN) criteria are 2 well-established systems used to estimate the risk of malignancy in GIST, and they all divide GIST into 4 groups (very low risk, low risk, intermediate risk, and high risk) based on pathological features, that is, tumor size, mitotic count, and tumor site. NIH criteria were often used in the included studies. Results showed that higher risk is associated with poorer rates of overall survival among patients with GIST.^[15]^ However, the clinical behaviors and outcomes of GIST still vary considerably, especially among patients in the high-risk category. It is important to objectively assess the biological behavior of GIST, which can become malignant; however, at present, this malignancy is difficult to determine histologically.

**Table 1:**
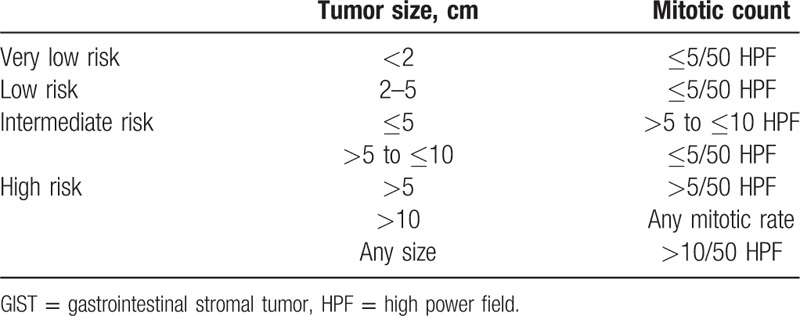
National Institutes of Health System of risk grading for GIST.

Ki67, a nuclear marker, exists in actively proliferating cells. It is expressed in all phases of the cell cycle in stages G1, S, and G2. It is considered a proliferation-related nuclear marker of tumor cells.^[16]^ The mitotic index reflects the M stage of mitosis only; however, as the Ki67 can recognize most of the proliferating cells except for G0, it is considered to be more appropriate as an marker of the malignancy of GIST.^[17]^ To date, a number of studies have reported a correlation between Ki67 expression and malignant risk of GIST.^[18–20]^ However, because of conflicting results and limited sample size, the significance of Ki67 in predicting risk of malignancy with GIST is still in dispute. In this study, we conducted a meta-analysis and attempted to explore the predictive value of Ki67 overexpression with respect to the risk of malignancy of GIST.

## 2 Methods

### 2.1 Publication search

The search of the literature was covered from Cochrane Library, PubMed, Medline, and Embase for studies published until October 31, 2016. Search terms included “gastrointestinal stromal tumor” OR “gastrointestinal stromal tumors” AND “Ki67.” Two investigators, CP and ZL, examined all the titles and the abstracts of the resulting articles. The first step was the selection of papers referring to the relationship between ki67 expression and the risk of malignancy in GIST. We then analyzed the full texts. We identified relevant trials from the reference list of each selected article. When multiple articles for a single study were available, only the most complete and current publication was used in this meta-analysis. Ethical approval and written informed consent of patients were not needed because the whole study was literature review and performed on the basis of previous researches.

### 2.2 Inclusion criteria

The inclusion criteria were as follows: articles evaluating Ki67 expression in GIST tissue as indicated by immunohistochemistry and biologic behavior; assessment by NIH risk system; and articles published in English on human subjects with the full text available for data retrieval before October 2016.

### 2.3 Exclusion criteria

The following articles were excluded: review articles without original data; original articles lacking the parameter of Ki67 or NIH/NCCN risk system or without clear incidence of Ki67 in NIH/NCCN system; and articles that dealt with cell lines or animals or case reports.

### 2.4 Data extraction

Two investigators (ZY and CP) independently assessed publications following the exclusion and inclusion criteria. Discrepancies between the 2 investigators were resolved by discussion with 2 senior authors (MA and LZ). The following information was then extracted from every study: first author's surname, publication date, method of categorization, total number of NIH risk groups, and population characteristics (age, sex, etc), incidence of Ki67 in NIH/NCCN risk groups. Minimum number was required for each study in our meta-analysis.^[21]^

### 2.5 Statistical analysis

All the statistical tests were performed with Review Manager Version 4.2 (The Cochrane Collaboration, Oxford, England), and statistical significance was set at *P* < .05. In the meta-analysis, heterogeneity assumption was calculated using the χ^2^-based Q test. A *P* value >0.1 for the Q test indicates a lack of heterogeneity among studies. The odds ratio (OR) estimate of each study was calculated using the fixed-effects model (the Mantel-Haenszel method). Otherwise, the random-effects model (the DerSimonian and Laird method) was used. The significance of the pooled OR was determined using the Z test and *P* >.05 was considered statistically significant. Sensitivity analyses were carried out to determine whether modification of the inclusion criteria of this meta-analysis affected the final results. Pooled ORs and 95% confidence intervals (CIs) were calculated for the dichotomous outcome data. An estimate of potential publication bias was carried out using the funnel plot. The asymmetry of the plot suggested publication bias. Funnel plot asymmetry was assessed using Egger linear regression test, a linear regression approach to measure funnel plot asymmetry on the natural logarithm scale of the OR. The significance of the intercept was determined using the *t* test, as suggested by Egger (*P* <.05 was considered representative of statistically significant publication bias).

## 3 Results

### 3.1 Characteristics of studies

**Figure 1:**
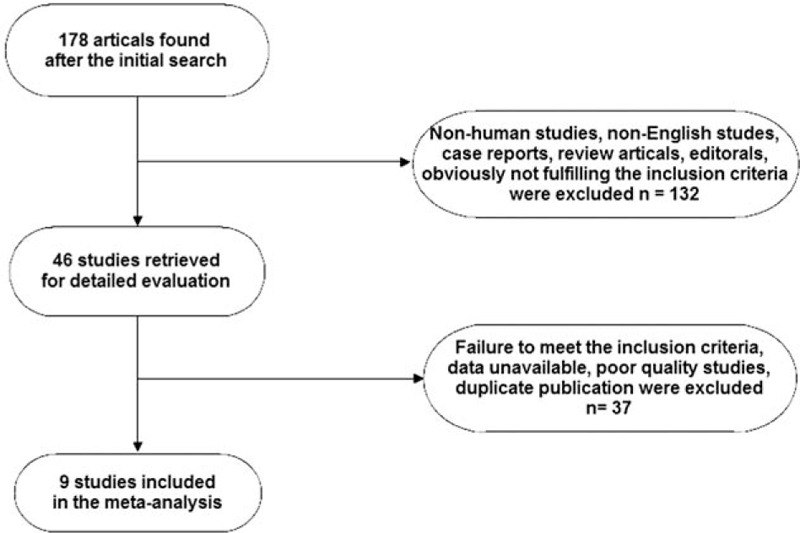
Flow chart of screening strategy for included studies.

After review of titles and abstracts, several studies were excluded because they did not contain sufficient information to calculate OR (Fig. 1). Finally, 9 studies that satisfied the inclusion criteria were included.^[17,22–29]^ The main features of the included studies are listed in Table 2. A total of 982 cases were included for meta-analysis.

**Table 2:**
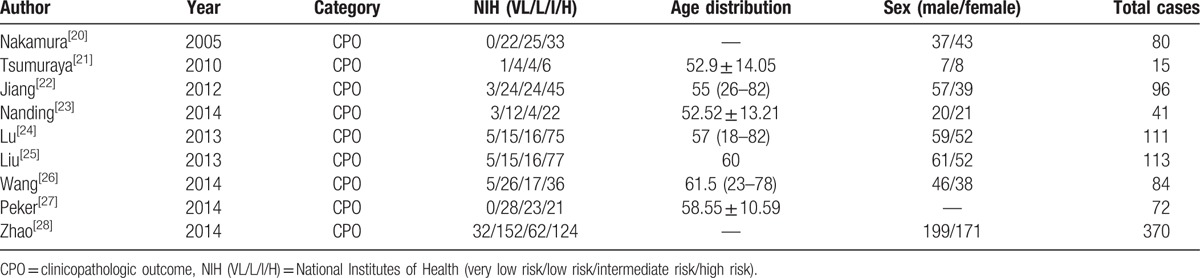
Main characteristics of all studies included in the meta-analysis.

### 3.2 Meta-analysis

**Figure 2:**
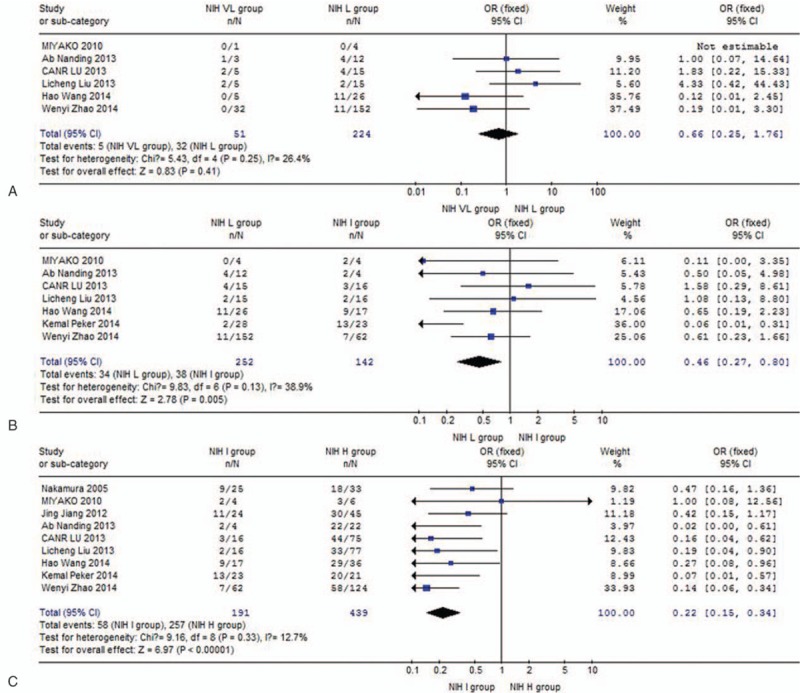
Meta-analysis of incidence of Ki67 overexpression among NIH subgroups: (A) NIH VL group versus NIH L group; (B) NIH L group versus NIH I group; (C) NIH I group versus NIH H group. H = high risk, I = intermediate risk, L = low risk, NIH = National Institutes of Health, VL = very low risk.

This meta-analysis showed that the incidence of Ki67 overexpression did not differ between the NIH VL group and NIH L group (OR: 0.66, 95% CI: 0.25–1.76; *P* = .41, *P*_heterogeneity_ = .25) (Fig. 2A). The incidence of Ki67 overexpression was significantly higher in the NIH I group than in the NIH L group (OR: 0.46, 95% CI: 0.27–0.80; *P* = .005, *P*_heterogeneity_ = .13) (Fig. 2B). Similarly, the incidence of Ki67 overexpression in NIH I group was higher than in the NIH H group (OR: 0.22, 95% CI: 0.15–0.34; *P* < .00001, *P*_heterogeneity_ = .33) (Fig. 2C). The test of heterogeneity for the 9 combined studies did not meet any single heterogeneity criterion (*P* > .05). No other single study affected the pooled OR qualitatively, as indicated by sensitivity analyses (data not shown).

### 3.3 Publication bias

**Figure 3:**
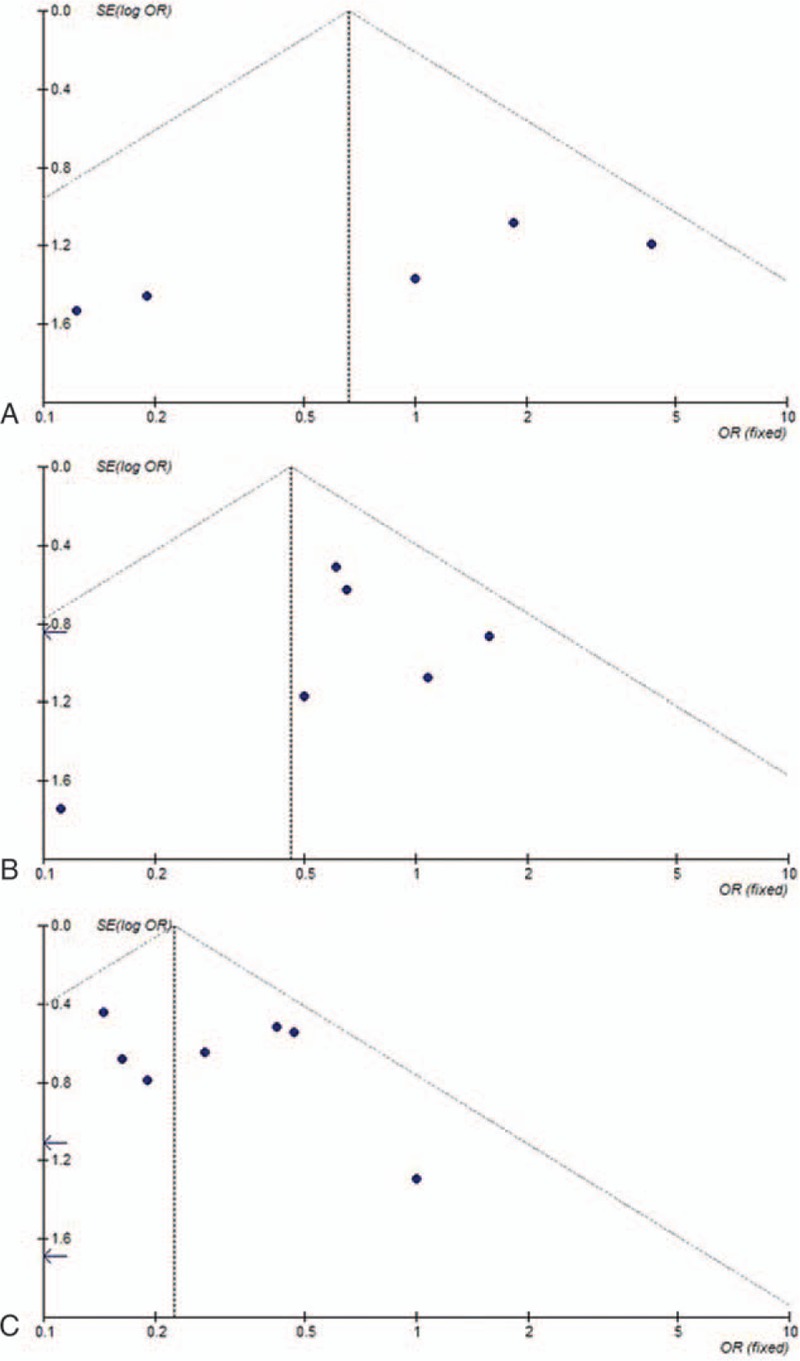
Begg funnel plot for publication bias test: (A) NIH VL group versus NIH L group; (B) NIH L group versus NIH I group; (C) NIH I group versus NIH H group. H = high risk, I = intermediate risk, L = low risk, NIH = National Institutes of Health, VL = very low risk.

A funnel plot was drawn to evaluate possible publication bias. The shapes of the funnel plots did not show any evidence of obvious asymmetry (Fig. 3A–C).

## 4 Discussion

Previous studies have reported that tumor size, cell count, mitotic index, and tumor location are useful in predicting GIST risk for malignant transformation.^[30–32]^ According to these clinicopathological parameters, NIH and NCCN systems have been established to predict GIST behavior by using risk assessment (very low risk, low risk, intermediate risk, and high risk). In this study, we conducted a meta-analysis to evaluate whether internal molecular events, especially ki67, are also correlated with the malignant risk in GIST.

Ki67, also known as MKI67, is present in actively proliferating cells in the G1, S, and G2 phases, and is a proliferation-related nuclear marker of tumor cells. A recent study has demonstrated that the automated assessments of Ki67 staining with computing image analysis can be used for prognostic assessments of patients with breast cancer.^[33]^ Several studies demonstrated that Ki67 is useful in predicting the malignant potential of GIST.^[20,34–37]^ Some studies have shown that Ki67 defines proliferation of cells in G1, S, and G2 phases, and therefore can be used as an objective criterion in the evaluation of GIST malignancy.^[38,39]^ Gumurdulu et al found that Ki67 is useful as a prognostic factor alongside tumor grade, tumor size, and the mitotic index.^[40]^ Current publications also suggest that a high Ki67 index may indicate metastasis and recurrence.^[41,42]^ Wong et al found that Ki67 was less reliable than the mitotic count, although it proved useful in assessing the proliferation rate of the tumor cells in GIST.^[43]^ The study suggested that the 1-, 3-, and 5-year survival rates of the Ki67 + GIST group were lower than those of the Ki67 group. Demir et al did not find any correlation between Ki67 overexpression and mitotic activity in tumors.^[44]^

Because of the conflicting results, we decided to investigate the correlation of Ki67 overexpression with malignant risk of GIST using meta-analysis. This study clearly showed greater rates of Ki67 overexpression in NIH-intermediate and NIH-high GIST risk groups. Interestingly, results did not show any significant difference in the incidence of Ki67 overexpression between the NIH VL and NIH L groups, suggesting that this is a process from quantitative change to qualitative change. The present study demonstrates that Ki67 expression may be an effective complement to the NIH criteria for predicting the risk of malignant GIST, especially for intermediate- and high-risk cases. As for the therapeutic implication of evaluating Ki67 index, Zhao et al. found Ki67 index >8% may be a negative factor to imatinib adjuvant therapy.^[29]^ In a previous study, we found KIT mutation in high-risk and malignant GIST to indicate poor prognosis, but patients with KIT mutation benefit from the targeted therapy of imatinib.^[8]^ It is here speculated that Ki67 expression and KIT mutation are both important independent prognostic markers of GIST. In this way, further multicenter and prospective studies with larger sample size are required to explore the internal correlation of Ki67 and KIT mutation in predicting the response to imatinib and use in clinical work.

The small sample size, varied clones of antibodies tested, and potential heterogeneity limit our ability to draw precise conclusions. More work is still needed to verify these results. Mitotic count and tumor size are still the most important prognostic criteria for classification of GIST. These, in conjunction with Ki67 index may be important to the prediction of the risk of malignancy in GIST, especially for intermediate- and high-risk NIH groups.
